# Dynamics of blood microsatellite instability (bMSI) burden predicts outcome of a patient treated with immune checkpoint inhibitors: a case report of hyperprogressive disease

**DOI:** 10.3389/fimmu.2025.1492296

**Published:** 2025-02-05

**Authors:** Daria Kravchuk, Alexandra Lebedeva, Olesya Kuznetsova, Alexandra Kavun, Anastasiia Taraskina, Ekaterina Belova, Tatiana Grigoreva, Egor Veselovsky, Vladislav Mileyko, Vladislav Nikulin, Lidia Nekrasova, Alexey Tryakin, Mikhail Fedyanin, Maxim Ivanov

**Affiliations:** ^1^ Moscow Multidisciplinary Clinical Center “Kommunarka” of the Department of Health of the City of Moscow, State Budgetary Institution of Healthcare, Moscow, Russia; ^2^ R&D Department, OncoAtlas LLC, Moscow, Russia; ^3^ Institute for Personalized Oncology, Sechenov First Moscow State Medical University, Moscow, Russia; ^4^ N.N. Blokhin Russian Cancer Research Center, Moscow, Russia; ^5^ Faculty of Physics, Lomonosov Moscow State University, Moscow, Russia; ^6^ P. Hertsen Moscow Oncology Research Institute (MORI), Moscow, Russia; ^7^ Federal State Budgetary Institution “National Medical and Surgical Center named after N.I. Pirogov” of the Ministry of Health of the Russian Federation, Moscow, Russia

**Keywords:** colorectal cancer, liquid biopsy, hyperprogression, next-generation sequencing, dMMR

## Abstract

Microsatellite instability (MSI) is a widely studied molecular signature, which is associated with long-term benefit in patients treated with immune checkpoint inhibitor therapy. This approach has been proven to be effective in the treatment of patients with MSI-positive colorectal cancer (CRC). Analysis of serial liquid biopsy samples allows to detect changes in the tumor in response to therapy. Typically, somatic mutations are used for tracing the dynamics of the tumor, and the assessment of DNA signatures such as MSI is not currently used for these purposes. Here, we describe a case of a MSI-positive CRC, who received nivolumab monotherapy. Sequential sampling of the patient’s plasma demonstrated an increase in MSI burden (bMSI), which was found to correlate with the increase of driver mutation burden one month after starting nivolumab, and hyperprogressive disease. Thus, analysis of bMSI in liquid biopsy via NGS may be a promising method for timely assessment of the treatment effectiveness received by patients with MSI-positive CRC.

## 1 Introduction

Microsatellite instability (MSI) is a phenomenon of hypermutation occurring at microsatellite loci, which are tracts of short repetitive DNA motifs. MSI comes as the shortening of the mononucleotide tracts due to failure to correct DNA polymerase slippage mistakes due to defects in the mismatch repair system (dMMR) (1, 2). MSI/dMMR occurs among various tumor types (3), and is associated with improved outcomes of patients treated with immune checkpoint inhibitors (ICI) (4–6). The effectiveness of ICI has been well demonstrated for patients with MSI-positive colorectal cancer (CRC) in numerous clinical studies (7–12). Standard diagnostic methods such as immunohistochemistry (IHC) or polymerase chain reaction (PCR) are routinely used in clinical practice for the assessment of MSI/dMMR and provide a binary assessment of MSI/dMMR (positive or negative) (13). Next generation sequencing (NGS) is an emerging method for assessing MSI (13–15). The advantages of assessing MSI status using NGS include the ability to study a larger number of short tandem repeats (STRs), as well as to simultaneously analyze the mutational profile and mutational burden (TMB) of the tumor (13). Much like routine methods, standard NGS pipelines classify tumors as stable or unstable based on the percentage of unstable STRs (14). Recent studies have demonstrated excellent sensitivity of NGS-based assays for MSI analysis in liquid biopsies (16). However, current binary classification of STRs only allows to analyze the percentage of unstable STRs. This approach does not allow for a quantitative assessment of MSI burden as the percentage of unstable DNA fragments in the sample (17). Similarly to the analysis of variant allele frequencies of mutations, the quantitative assessment of MSI might reflect the clonality of the tumor, as well as can be utilized to measure tumor dynamics when liquid biopsy is used. The use of liquid biopsy makes it possible to detect early changes in response to therapy that cannot be detected by standard imaging methods such as computer tomography (CT) (18–22). Typically, somatic mutations are used for tracing the dynamics of the tumor. However, the assessment of DNA signatures such as MSI is not currently used for these purposes. Here, we report a clinical case of a colorectal cancer patient with MSI confirmed by PCR and NGS. The patient was treated with ICI, eventually demonstrating hyperprogression. An algorithm for qualitative measurement of MSI burden was used for tracking its changes in serial on-treatment plasma samples (bMSI). Here, for the first time, we demonstrate that the dynamics of bMSI correlate with driver mutational burden and disease progression.

## 2 Case presentation

A 40-year-old male was diagnosed with sigmoid cancer in November 2021. He underwent right-sided hemicolectomy on 23 November 2021. Histologically, colon adenocarcinoma pT3N1bM0 was then confirmed. CT scan in December 2021 revealed no signs of continued tumor growth and a lesion in upper lobe (S4) of the right lung up to 18 mm with no enlargement in size compared to previous CT scans. The patient then underwent 6 cycles of adjuvant chemotherapy with XELOX and 3 cycles of capecitabine monotherapy from December 2021 to July 2022. In January 2022, in between treatment cycles, the patient underwent PET/CT scan, which did not confirm a lesion in the lung as metastatic due to low 18FDG uptake. In April 2022, no KRAS/NRAS/BRAF alterations were found via PCR, and the IHC for HER2 was negative. However, following the results of a 5-loci PCR, the tumor was found to be MSI-positive. In line with the patient’s decision, no subsequent germline testing to rule out Lynch syndrome was performed. Following the completion of adjuvant chemotherapy, the patient underwent PET/CT in August 2022, which revealed the enlargement of size and contrast accumulation in S4 of the right lung (up to 5 cm) and the appearance of a new lesion, measuring up to 3 cm in the right hepatic lobe (S8). As it was not clear whether an inflamed retention cyst or metastatic lesion was observed in the lung, a bronchoscopy with brush-biopsy was performed. Consequent cytology did not reveal any atypical cells, and the lesion was determined a cyst. The patient was offered to undergo a liver biopsy to confirm the progression of the disease, but he declined and decided to remain under observation.

**Figure 1:**
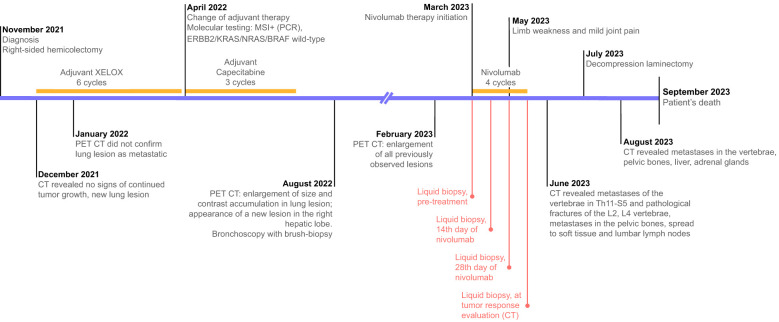
Treatment timeline and diagnostic procedures. Text in red reflects the timeline of the collection of serial liquid biopsy samples.

**Figure 2:**
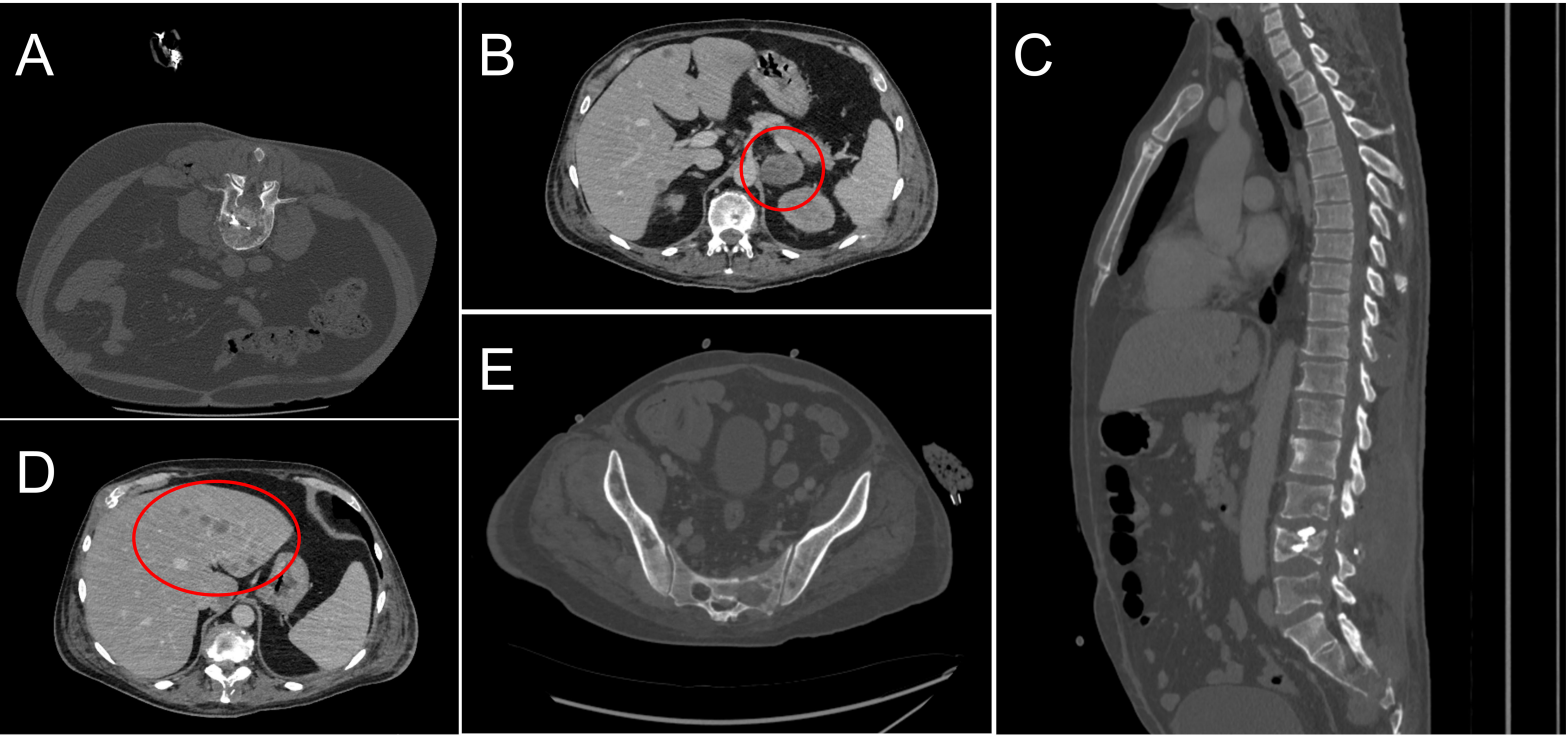
Computed tomography scans of the patient’s metastatic sites in the course of the treatment. **(A)** Metastasis and pathologic fracture in L4 revealed in June 2023; **(B)** Metastatic lesions in the adrenal glands, August 2023; **(C)** Metastatic lesions in the Th11-S5 vertebrae, June 2023; **(D)** Metastatic lesions in the liver, August 2023; **(E)** Destruction of pelvic bones, August 2023.

The next follow-up visit was in February 2023. The patient underwent CT that revealed the same lesions: 55x36 mm in S4 of the right lung and 34x27 mm in the S8 of the liver. Due to the enlarged size of the lesions, the patient was offered to undergo systemic therapy first. As the primary tumor was MSI-positive, monotherapy with nivolumab was recommended. The patient was then enrolled in an observational clinical trial BLOOMSI (NCT06414304). As part of the trial, the pre-treatment blood plasma sample was collected on 27.03.2023. On March 28th 2023, the patient started nivolumab. During the time on treatment, two serial plasma samples were collected, 17 and 31 days after therapy initiation (on 13.04.2023 and 27.04.2023, respectively). On May 2nd 2023, while receiving the 3rd cycle, the patient complained of limb weakness and mild joint pain. Following the recommendation of a general practitioner, he started to take еtoricoxib with short term pain relief. The patient received a total of 4 cycles of nivolumab, and the symptoms were increasing. After the discontinuation of nivolumab, a post-treatment blood plasma sample was obtained (on 11.05.2023, 45 days after the start of treatment). In the beginning of June, patient underwent radiography of the hip joints and CT of the brain, but no abnormalities were found. Further CT scan of the lumbosacral region of the spine revealed metastases of the vertebrae in Th11-S5 and pathological fractures of the L2, L4 vertebrae bodies. Metastases were also found in pelvic bones leading to their destruction, with the spread of the pathological process to soft tissue at the level of the iliac crests and lumbar lymph nodes. Furthermore, adrenal glands and liver were affected by metastatic lesions. Decompression laminectomy at the level of L4 vertebrae with tumor removal with microsurgical reconstruction of the L4 root nerve with posterior stabilization was performed on July 7th. Subsequent histology confirmed the colorectal origin of the metastatic lesion. Following the surgery, the patient did not receive any systemic treatment. In August 2023, he received treatment for lumbosacral abscess after laminectomy and died a month later (**Figures 1**, **2**).

## 3 Genomic testing and bMSI burden dynamics

Consistent with current treatment guidelines, MSI was evaluated via standard 5-loci PCR panel, uncovering MSI positivity in the patient’s primary tumor sample. Based on the results of PCR, the patient was included in the local observational clinical trial evaluating the dynamics of MSI burden in serial samples from patients receiving ICI. As part of the trial, confirmatory MSI/dMMR testing was performed using a 4-antibody IHC (loss of two proteins was observed) and NGS via Solo test Atlas Pro panel covering 34 commonly altered cancer-related genes and 30 MSI mononucleotide tandem repeats. NGS was performed on primary tumor and serial plasma (liquid biopsy, LB) samples received prior to the start of ICI therapy, on the 17th, 31st days on therapy, as well as after the discontinuation of ICI.

**Figure 3:**
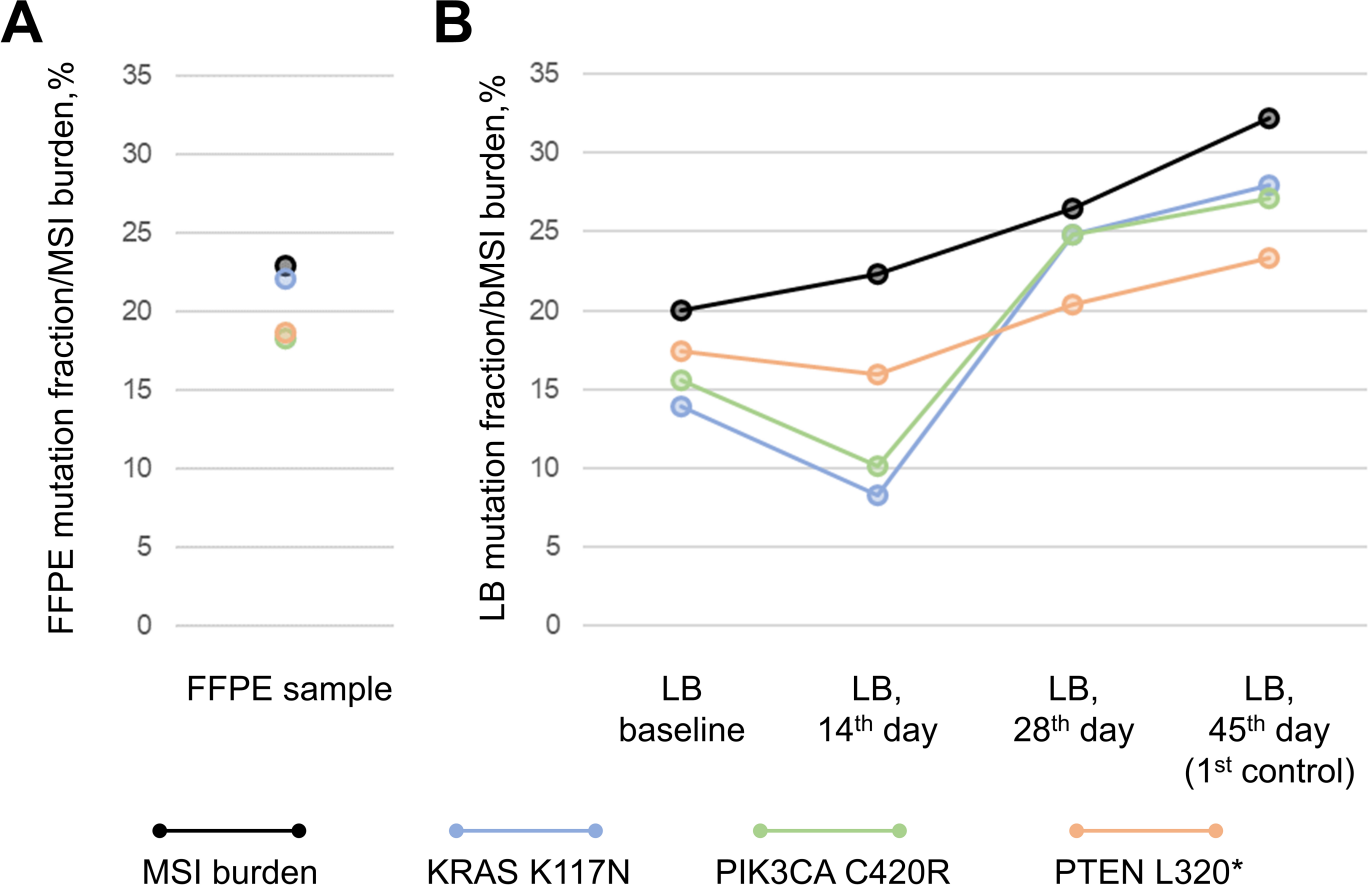
Molecular profile of FFPE sample **(A)** and dynamics of driver mutations and DNA signatures during the course of immunotherapy as traced via serial liquid biopsy samples (LB) **(B)**. *premature stop-codon.

NGS revealed point mutations in KRAS, PIK3CA, PTEN and TP53 (the latter was only observed in the FFPE sample) in the pre-treatment FFPE and LB samples (**Figure 3**). Of note, initial PCR testing for common CRC alterations in colorectal cancer found no KRAS mutations, whereas NGS testing of a tumor sample found a rare hotspot KRAS p.Lys117Asn mutation (23) with a VAF of 22%. MSIsensor2 (14) revealed a slight decrease unstable STRs by 3% at the 14th day after therapy initiation and gradual increase by 7% and 12% in the subsequent liquid biopsy samples during ICI treatment (73%, 71%, 76% and 85% for baseline, 14th day, 28th day and 1st control LB, respectively). KRAS, PIK3CA and PTEN driver mutations were identified in LB samples with almost the same variant allele frequency (13.9, 15.6 and 17.3% respectively) (**Table 1**), similarly to the molecular profile seen in primary FFPE sample (22.1%, 18.2% and 18.7%, respectively) alluding to its homogeneous representation across tumor clones in primary tumor. Across serial LB samples driver mutations demonstrated positive dynamics at 14th day on therapy (decrease by 41%, 35% and 8% for KRAS, PIK3CA and PTEN mutations, respectively), and negative dynamics afterwards, although PTEN mutation demonstrated dissimilar dynamics contrasting to ones of PIK3CA and KRAS hinting at the positive selection of PTEN-mutated clone during the treatment (**Figure 3B**).

**Table 1:** Point mutations observed in the patient’s FFPE and serial plasma samples.

Variant	Driver/passenger	Pre-treatment FFPE (VAF, %)	Pre-treatment LB (VAF, %)	LB, 14th day of Nivolumab (VAF, %)	LB, 28th day of Nivolumab (VAF, %)	LB, tumor response assessment (VAF, %)
KRAS p.Lys117Asn	Driver	22.09%	13.90%	8.24%	24.79%	27.98%
PIK3CA p.Cys420Arg	Driver	18.25%	15.56%	10.11%	24.82%	27.16%
PTEN p.Leu320Ter	Driver	18.69%	17.38%	15.92%	20.33%	23.35%
TP53 p.Pro151Leu	Driver	20.30%	0%	0%	0%	0%
H3-3A p.Ala22LeufsTer15	Passenger	0%	0%	4.65%	0%	0%
RAF1 p.Phe240Cys	Passenger	0%	0%	4.15%	0%	0%
FGFR2 p.Gly543Arg	Passenger	0%	0%	0%	3.04%	0%
STK11 c.374 + 5C>T	Passenger	0%	0%	0%	5.72%	0%
CYP2D6 p.Pro35Ser	Passenger	0%	0%	0%	3.81%	0%
RAF1 p.Val266Ile	Passenger	0%	0%	0%	4.71%	0%
PIK3CA p.Asp538Asn	Passenger	0%	0%	0%	3.41%	0%
MET p.Pro173Ser	Passenger	0%	0%	0%	3.74%	0%
MET p.Ile1102Leu	Passenger	0%	0%	0%	5.78%	0%

VAF, variant allele frequency. The alterations were classified as driver or passenger based on the published evidence (24, 25).

To assess MSI quantitatively in FFPE sample, i.e. to estimate the percentage of tumor cells exhibiting MSI, we calculated the ratio of total amount of sequencing reads in NGS data supporting DNA fragments with altered STR sequences (i.e. demonstrating shortage of reference STR length by 4b.p. and more) to the total amount of sequencing reads supporting any STR sequences. The calculated percentage was close to the VAF of KRAS, PIK3CA and PTEN mutations (**Figure 3A**), suggesting that quantitatively estimated MSI burden was correlated with tumor clonality. Similarly to the FFPE sample, MSI burden was in line with VAFs of driver mutations in pre-treatment LB sample. Further tracing of bMSI burden across serial LB samples demonstrated a gradual increase without significant drop-outs seen for KRAS and PIK3CA mutations hinting at co-evolution of tumor clones exhibiting PTEN mutation and MSI.

## 4 Discussion

MSI/dMMR is a known biomarker of ICI response across tumor types, including colorectal cancer (26, 27). Gold standard methods for MSI/dMMR detection include PCR and IHC, however in recent years NGS has been recognized as a potentially decisive tool for selecting candidates for ICI (13). Although tumor samples are considered preferential for MSI/dMMR testing, various issues, including insufficient tumor purity and overall suboptimal tissue quality might result in ambiguous results (28). Large-scale studies suggest that liquid biopsy might perform as well as tumor samples for identifying MSI-positive patients who are candidates for ICI (20, 29, 30).

A high concordance was observed between baseline tissue and plasma mutations (**Table 1**). Evaluation of MSI dynamics in liquid biopsies has been reported to correlate with response to immunotherapy or lack thereof (31, 32). Additionally, serial ctDNA analysis has been shown to be prognostic of tumor dynamics, with an increase of ctDNA indicating progressive disease (33–36). In our patient, while an increase in MSI burden was immediate and persistent, the increase of driver and passenger mutational burden was delayed in both pre- and on-treatment serial samples. Moreover, bMSI increase preceded the first clinical signs of disease progression, whereas an increase in mutational burden coincided with disease progression (**Figure 3**). Since no CT imaging was done in the course of nivolumab treatment, we could not compare the results of imaging studies to LB analysis, however, the results of the latter are consistent with the patient’s symptoms, which were later confirmed to have originated from progressive disease. Previous studies have suggested that analysis of LB has the potential to outperform standard radiographic imaging for predicting treatment outcomes (37), however no studies have reported the utility of MSI burden as a screening tool for monitoring tumor dynamics in the course of ICI. While these findings are interesting, they require further validation in prospective clinical trials to validate the role of bMSI for tumor response monitoring in MSI-positive patients.

It is worth mentioning that initial tumor testing with PCR, in contrast with NGS, did not reveal a KRAS p.Lys117Asn hotspot mutation (23). Precise determination of KRAS mutational status is crucial for tailoring therapy for patients with advanced MSS/pMMR colorectal cancer (38). Although seen at lower rates than mutations affecting codons 12, 13 and 61, the KRAS codon 117 mutation is considered a ‘standard’ KRAS mutation, indicating that all standard testing methodologies used in the clinic should be able to detect this mutation. For instance, in KEYNOTE-177, no benefit from pembrolizumab monotherapy was observed for patients with MSI and RAS mutations (11). However, as seen in the CheckMate 8HW trial, the presence of RAS mutations does not interfere with the efficacy of anti-PD-1 and anti-CTLA-4 combination therapy for patients with MSI-positive CRC (39), suggesting that dual ICI might have been preferential for this patient. Results of these studies suggest that oncogenic KRAS mutations have a potential to influence the patient’s outcome following nivolumab monotherapy. However, this was not observed in our case, as we saw significant degression of KRAS-mutated clones at day 14 after therapy initiation in contrast to tumor clones possessing PTEN-mutation and MSI.

The term hyperprogression has been a topic of debate among the scientific community, however since it indicates the state of rapid progression of disease in the course of immunotherapy, it seems applicable for our patient’s case (40). Hyperprogression in response to immunotherapy is often linked to MDM2 or MDM4 amplification, however, targeted NGS sequencing performed for our patient did not allow for MDM2/4 analysis, since these genes were not included in the panel design (41). Furthermore, the small panel size did not allow for the evaluation of tumor mutational burden (TMB). TMB is an important predictive biomarker of ICI benefit, reflecting a number of mutations occurring in tumor cells. Tumors with high TMB tend to be more immunogenic and more responsive to ICI therapy (42). Even in the presence of MSI, patients with low TMB demonstrate poor outcomes when treated with ICI (43). However, given the relatively low amount of passenger mutations observed in our patient’s samples, ultra-high TMB could be potentially ruled out, as in samples with ultra-high TMB harbor passenger mutations in at least one of the genes analyzed by our panel. For instance, 60% and 76% of patients included in the MSK MetTropism study with TMB>20 Mut/Mb and >40 Mut/Mb, respectively, had at least one passenger mutation in the analyzed genes (44). Additionally, some authors have linked EGFR amplifications to the state of hyperprogression in response to immunotherapy (40), however, our patient was EGFR amplification-negative. However, since the information on MDM2/4/EGFR amplifications was unavailable due to the panel design, the question of whether the patient’s hyperprogressive disease was associated with molecular correlates. Although high TMB could be potentially ruled out in this case, precise TMB estimation could not be performed, which could be considered a significant limitation of the study, since TMB is an important biomarker of ICI benefit. Moreover, the activation of T-helpers has gained attention as another mechanism of ICI resistance (45). Finally, 30-40% of patients treated with ICI fail to respond to therapy (46).

Overall, our patient’s case highlights the potential of ctDNA MSI burden as a tool for monitoring tumor response and clonal evolution in the course of ICI in patients with MSI-positive colorectal cancer. Further study of the clinical application of MSI burden by liquid biopsy could potentially allow timely assessment of treatment effectiveness in patients before visible signs of progression over time among patients with colorectal and other MSI-positive solid tumors.

## Data Availability

The raw data supporting the conclusions of this article will be made available by the authors, without undue reservation.
